# Dimethyl fumarate blocks pro-inflammatory cytokine production via inhibition of TLR induced M1 and K63 ubiquitin chain formation

**DOI:** 10.1038/srep31159

**Published:** 2016-08-08

**Authors:** Victoria A. McGuire, Tamara Ruiz-Zorrilla Diez, Christoph H. Emmerich, Sam Strickson, Maria Stella Ritorto, Ruhcha V. Sutavani, Anne Weiβ, Kirsty F. Houslay, Axel Knebel, Paul J. Meakin, Iain R. Phair, Michael L. J. Ashford, Matthias Trost, J. Simon C. Arthur

**Affiliations:** 1 Division of Cell Signaling and Immunology, School of Life Sciences, Wellcome Trust Building, University of Dundee, Dow St, Dundee, DD1 5EH, UK; 2 Department of Chemistry and Biochemistry, Faculty of Pharmacy, CEU San Pablo University, Urbanización Montepríncipe, 28668 Madrid, Spain; 3 MRC Protein Phosphorylation and ubiquitylation Unit, School of Life Sciences, Sir James Black Centre, University of Dundee, Dow St, Dundee, DD1 5EH, UK; 4 Cardiovascular and Diabetes Medicine, Medical Research Institute, School of Medicine, University of Dundee, Ninewells Hospital, Dundee, DD1 9SY, UK

## Abstract

Dimethyl fumarate (DMF) possesses anti-inflammatory properties and is approved for the treatment of psoriasis and multiple sclerosis. While clinically effective, its molecular target has remained elusive - although it is known to activate anti-oxidant pathways. We find that DMF inhibits pro-inflammatory cytokine production in response to TLR agonists independently of the Nrf2-Keap1 anti-oxidant pathway. Instead we show that DMF can inhibit the E2 conjugating enzymes involved in K63 and M1 polyubiquitin chain formation both *in vitro* and in cells. The formation of K63 and M1 chains is required to link TLR activation to downstream signaling, and consistent with the block in K63 and/or M1 chain formation, DMF inhibits NFκB and ERK1/2 activation, resulting in a loss of pro-inflammatory cytokine production. Together these results reveal a new molecular target for DMF and show that a clinically approved drug inhibits M1 and K63 chain formation in TLR induced signaling complexes. Selective targeting of E2s may therefore be a viable strategy for autoimmunity.

Autoimmune disorders represent a diverse range of conditions that remain challenging to treat. The advent of biological drugs, such as anti-TNF agents, provided a significant advance in the treatment of these conditions^1^, however they have the disadvantages of not being orally available and that a proportion of patients do not respond. The development of new orally available small molecule drugs for autoimmunity is therefore desirable. Several breakthroughs in this area have recently been made, such as the development of Jak inhibitors and S1P receptor modulating agents, which illustrate the potential of this approach^2^,^3^,^4^.

Dimethyl fumarate (DMF) is a methyl ester known to have immuno-modulatory properties. In combination with other fumaric acid esters, DMF has been in use for many years as a treatment for moderate and severe psoriasis^5^. The first report of its use was in 1959^6^, although it did not gain widespread acceptance until some time later following the publication of the first clinical trials demonstrating its efficacy in 1990^7^. Subsequently, DMF in combination with three salts of ethylhydrogenfumarate was licensed for use in psoriasis in Germany in 1994^8^,^9^. More recently, a slow release formulation of DMF has been approved for the treatment of multiple sclerosis^10^.

The molecular target of DMF that accounts for its ability to modulate the immune system has been elusive. Amongst the possible explanations for its action, DMF has been shown to reduce T cell numbers, inhibit NFκB mediated transcription and activate the Nrf2 pathway (reviewed in^11^,^12^). In addition, DMF has been found to modulate cytokine production in a number of immune cell types: cytokine production is regulated by several intracellular signaling systems including NFκB and the ERK1/2 and p38 MAPK pathways, and DMF has been suggested to modulate these pathways. For example, DMF has been shown to prevent the induction of NFκB dependent transcription in LPS stimulated dendritic cells as well as TNF stimulated Human Umbilical Vein Endothelial Cells (HUVEC) or airway smooth muscle cells (ASMC)^13^,^14^,^15^.

The reported effects of DMF on MAPK signaling are less clear. While some studies have shown that DMF could decrease ERK1/2 activation in cells, others have found it to have no effect^14^,^16^,^17^. For p38, DMF has been reported to either have no effect on activation or to result in an increase in p38 phosphorylation^14^,^18^. MAPKs can, in part, mediate their cellular effects via the activation of downstream kinases. For example, p38α activates the downstream kinases MK2 and MK3 to promote the production of TNF^19^. In addition, p38α can also activate the kinases MSK1 and MSK2^20^. These two kinases, which can also be activated by ERK1/2^20^, have been found to have anti-inflammatory functions in macrophages and are required for the maximal induction of IL-10 by macrophages and dendritic cells^21^,^22^. The ERK1/2 pathway can also activate RSK^23^, however the role that this kinase plays in the regulation of cytokine production is less well established. DMF has been shown to affect the activation of both MSKs and RSKs^14^,^16^,^17^. For instance, in keratinocytes DMF selectively blocked MSK1 phosphorylation but not ERK1/2 or p38α activation in response to IL-1 stimulation^16^. Similarly DMF also blocked MSK1 and RSK activation in MIF (Macrophage Inhibitory Factor) stimulated keratinocytes and prevented the induction of Cox2^17^, a known MSK target gene^24^. DMF has also been reported to inhibit MSK1 phosphorylation in LPS stimulated dendritic cells, however in contrast to the data in keratinocytes, in dendritic cells DMF was able to reduce LPS induced ERK1/2, although not p38 or JNK, phosphorylation^14^.

In this study we examine the mechanism by which DMF blocks cytokine induction in primary macrophages and demonstrate that it affects signaling by inhibiting the formation of M1/K63 hybrid polyubiquitin chains.

## Results

### DMF inhibits the transcription of cytokines independently of Nrf2

**Figure 1:**
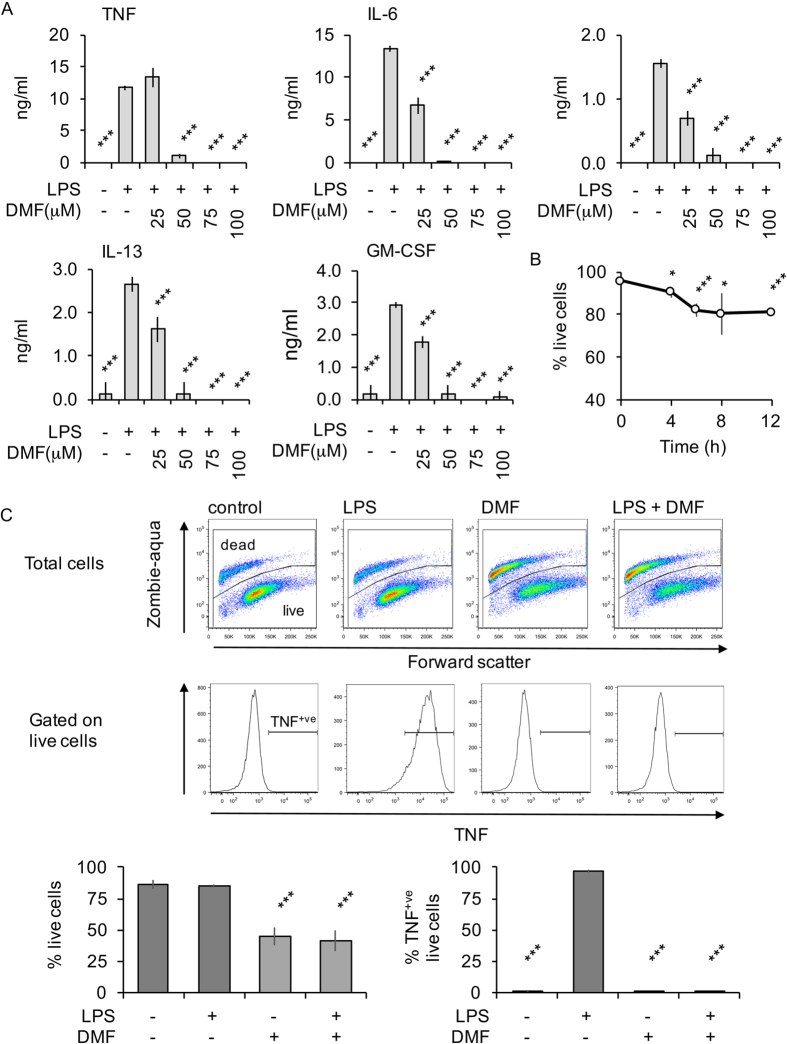
Inhibition of LPS stimulated cytokine induction by DMF. (**A**) BMDMs were incubated with 0, 25, 50, 75 or 100 μM DMF for 4 h. Cells were then stimulated with 100 ng/ml LPS for 8 h and the levels of TNF, IL-6, IL-10, IL-13 and GM-CSF determined. (**B**) BMDMs were incubated with 50 μM DMF for the indicated times and cell viability was determined by staining with Zombie-Aqua. Results show average and standard deviation of 4 independent treatments. (**C**) BMDMs were incubated in 50 μM DMF where indicated. Cells were then stimulated with 100 ng/ml LPS for 5 h. Cells were then stained for intracellular TNF levels and viability (Zombie-Aqua) as described in the methods. Representative stimulations are shown in the FACS plots while the graphs show average and standard deviation from 3 independent stimulations. In (**A,C**) a p value (Students t-test) of less than 0.001 relative to the LPS alone condition is indicated by ***.

**Figure 2:**
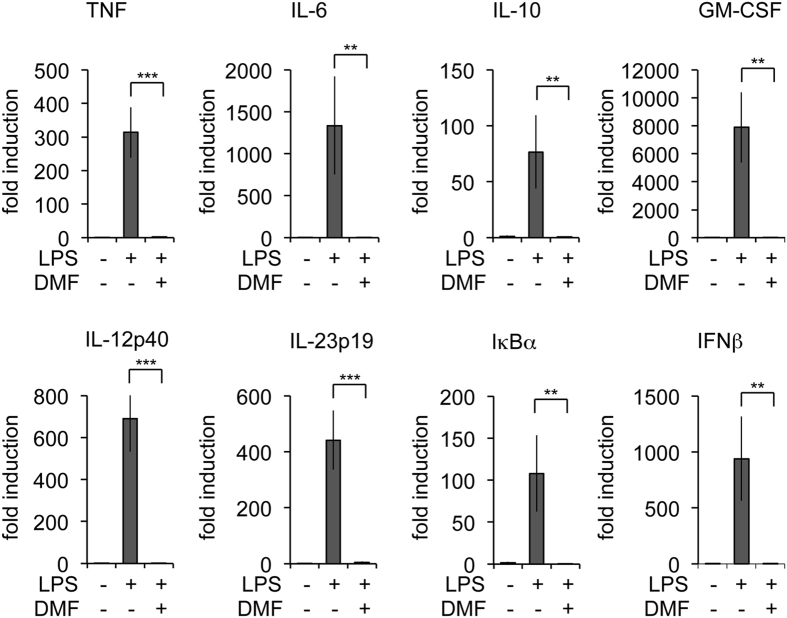
DMF inhibits LPS induced gene transcription. BMDMs were incubated with 50 μM DMF for 4 h and then stimulated with LPS for 1 h as indicated. Total RNA was then isolated and the induction of TNF, IL-6, IL-10, GM-CSF, IL-12p40, IL-23p19, IκBα and IFNβ determined by qPCR as described in the methods. Error bars represent the standard deviation of 4 BMDM cultures. For a comparison of LPS stimulation with or without DMF treatment a p value (Students t-test) of <0.01 is indicated by ** and p < 0.001 by ***.

To test the ability of DMF to block cytokine production in response to TLR agonists, BMDMs were incubated with various concentrations of DMF for 4 h (Fig. 1A). The cells were then stimulated with the TLR4 agonist LPS for a further 8 h and cytokine release determined. LPS promoted the secretion of TNF, IL-6, IL-10, IL-13 and GM-CSF; this was blocked by 50 μM DMF (Fig. 1A). To ensure this was not due to a loss of cell viability, cells were incubated with 50 μM DMF and viability determined by FACS. DMF did cause some cell death that increased over time, however the majority of cells were still alive following 12 h of DMF treatment (Fig. 1B). Cells were then treated with or without DMF in the presence of brefeldin A and monensin to block cytokine secretion. TNF levels were then measured at a single cell level by flow cytrometry following gating on the live cell population. This showed that DMF blocked LPS stimulated TNF production in the live cells (Fig. 1C). In line with the loss of cytokine secretion (Fig. 1A), DMF also repressed the induction of various cytokine mRNAs, including TNF, IL-6, IL-10, GM-CSF, IL-12p40, IL-23p19 and IFNβ, in response to 1 h of LPS stimulation (Fig. 2). DMF also suppressed the induction of IκBα mRNA, an established NFκB target gene.

**Figure 3:**
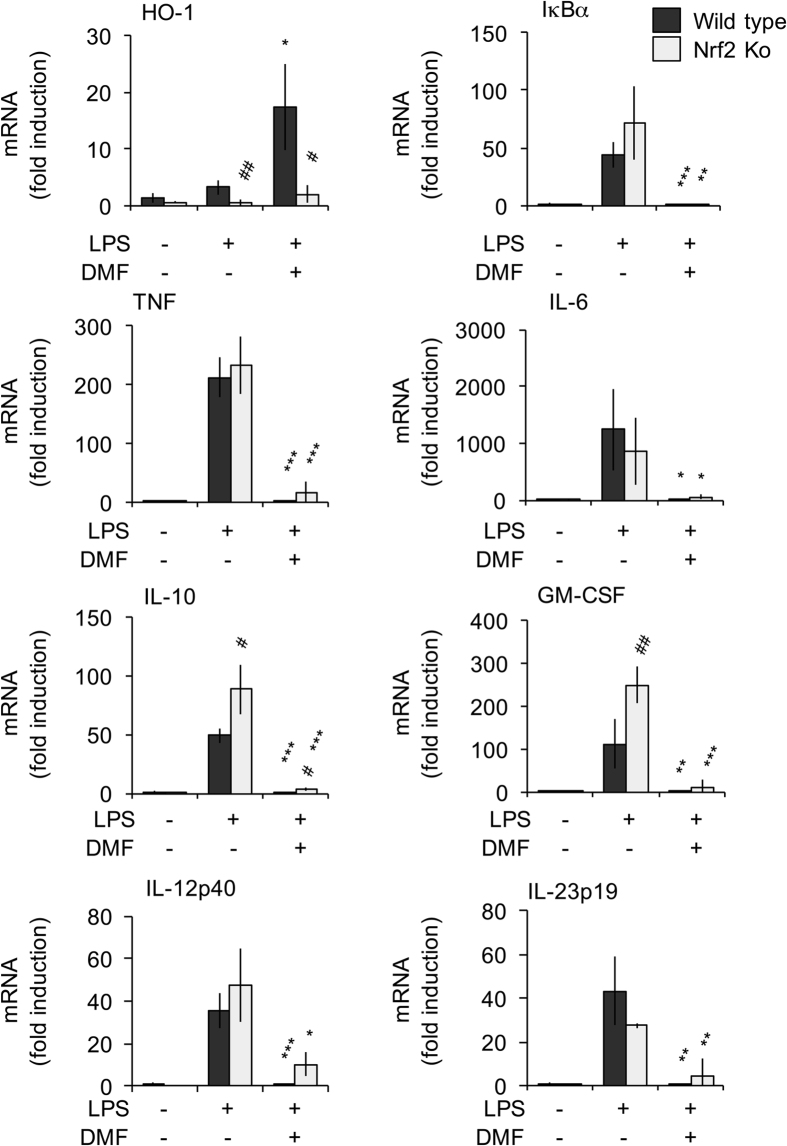
Effect of DMF on LPS stimulated mRNA induction in Nrf2 knockout BMDMs. BMDMs were isolated from wild type and Nrf2 knockout mice. Cells were incubated with 50 μM DMF for 4 h and then stimulated with LPS for 1 h as indicated. Total RNA was then isolated and the induction of TNF, IL-6, IL-10, GM-CSF. IL-12p40, IL-23p19, IκBα and HO-1 determined by qPCR. Error bars represent the standard deviation of measurements from independent cultures from 4 mice per genotype. For a comparison of LPS stimulation in the presence and absence of DMF a p value (Students t-test) of <0.05 in indicated by *, p < 0.01 by ** and p < 0.001 by ***. For a comparison between wild type and Nrf2 knockout cells a p < 0.05 is indicated by ^#^ and p < 0.01 by ^##^.

DMF has previously been suggested to act via targeting cysteine residues in Keap1 and activating the Nrf2 anti-oxidant pathway^25^,^26^,^27^. To test the potential involvement of this pathway in the regulation of cytokine transcription, Nrf2−/− BMDMs were stimulated with LPS in the presence or absence of DMF and cytokine mRNA levels measured at 1 h. Nrf2 regulates the transcription of several genes including HO-1^28^,^29^. While LPS alone did not induce the mRNA for HO-1, this mRNA was induced in DMF treated wild type but not Nrf2 knockout BMDMs, indicating that DMF was able to activate Nrf2 in macrophages (Fig. 3). Nrf2 knockout did not affect the induction of TNF, IL-6, IL-12p40, IL-23p19 or IκBα mRNAs in response to LPS relative to wild type cells. There was a small but significant increase (p < 0.05, Students unpaired two tailed t-test) in LPS stimulated IL-10 and GM-CSF mRNA induction in Nrf2 knockout cells compared to wild type BMDMs (Fig. 3). This increase in IL-10 mRNA induction is consistent with a recent report showing increased IL-10 production in Nrf2 knockout dendritic cells^30^. DMF was able to inhibit cytokine and IκBα mRNA induction in response to LPS in both wild type and Nrf2−/− cells (Fig. 3). These results suggest that the effects of DMF on LPS induced cytokine induction are largely independent of any effects on the Nrf2 pathway.

**Figure 4:**
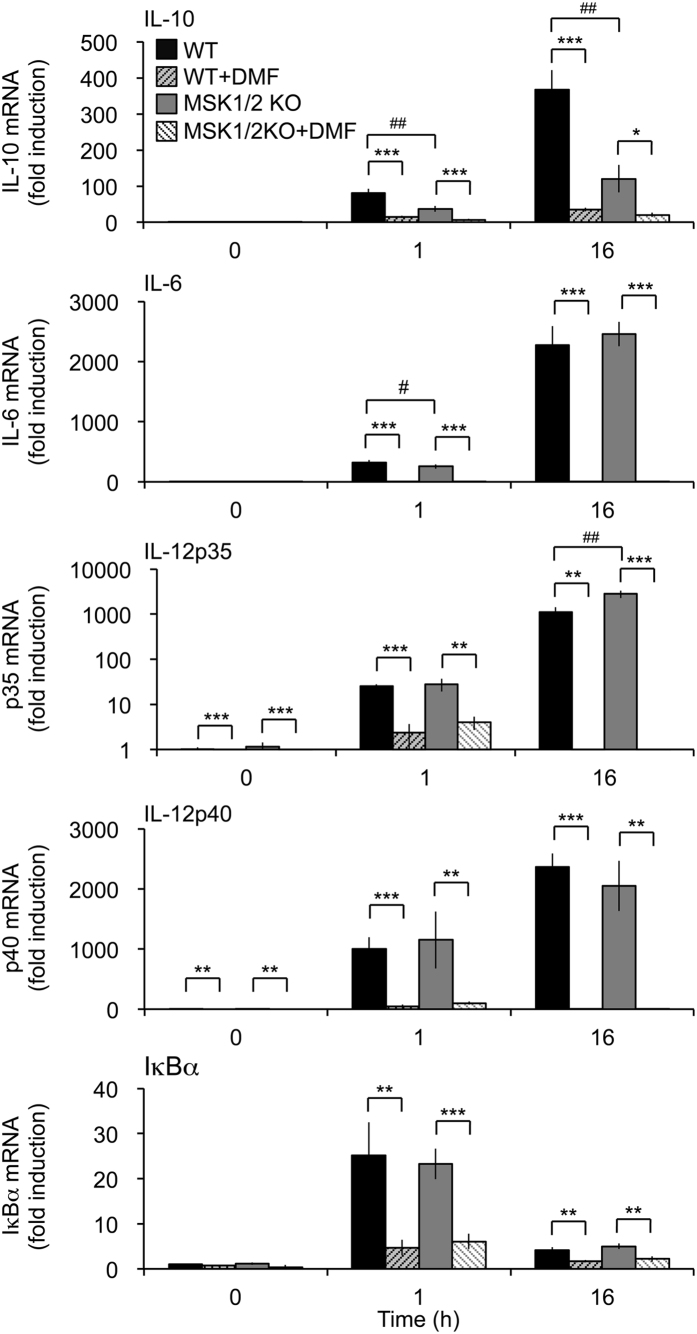
Effect of DMF on LPS stimulated mRNA induction in MSK1/2 knockout BMDCs. BMDCs were isolated from wild type and MSK1/2 double knockout mice. Cells were incubated with 50 μM DMF for 4 h and then stimulated with LPS for 1 h or 16 h as indicated. Total RNA was then isolated and the induction of IL-10, IL-6, GM-CSF, IL-12p35, IL-12p40 and IκBα was determined by qPCR. Error bars represent the standard deviation of measurements from independent cultures from 4 (0 and 1 h) or 3 (16 h) mice per genotype. A p value (Students t-test) between the presence and absence of DMF of less that 0.01 is indicated by ** and a p < 0.001 by ***. For a comparison of wild type and MSK1/2 knockout cells in the absence of DMF, p < 0.05 and p < 0.01 are indicated by ^#^ and ^##^ respectively.

DMF has been proposed to inhibit the action of MSK1 in dendritic cells^14^. However, the reduced cytokine production caused by DMF in Fig. 1 is inconsistent with the decreased IL-10 production but increased pro-inflammatory cytokine production previously reported in MSK1/2 double knockout macrophages^22^, suggesting that DMF must have MSK independent effects. To confirm this, wild type and MSK1/2 knockout BMDCs were tested. As expected MSK1/2 knockout BMDCs exhibited a lower induction of the MSK1/2 target gene IL-10 relative to wild type cells (Fig. 4). Induction of IL-12p35 mRNA was increased in the MSK1/2 knockout BMDCs while IL-6, IL-12p40 and IκBα mRNA induction was not greatly changed. As in macrophages, DMF blocked the LPS induced expression of both IκBα and the cytokine mRNAs tested. This inhibition was comparable in wild type and MSK1/2 knockout cells (Fig. 4).

### DMF inhibits NFκB and ERK1/2 activation in response to TLR agonists

**Figure 5:**
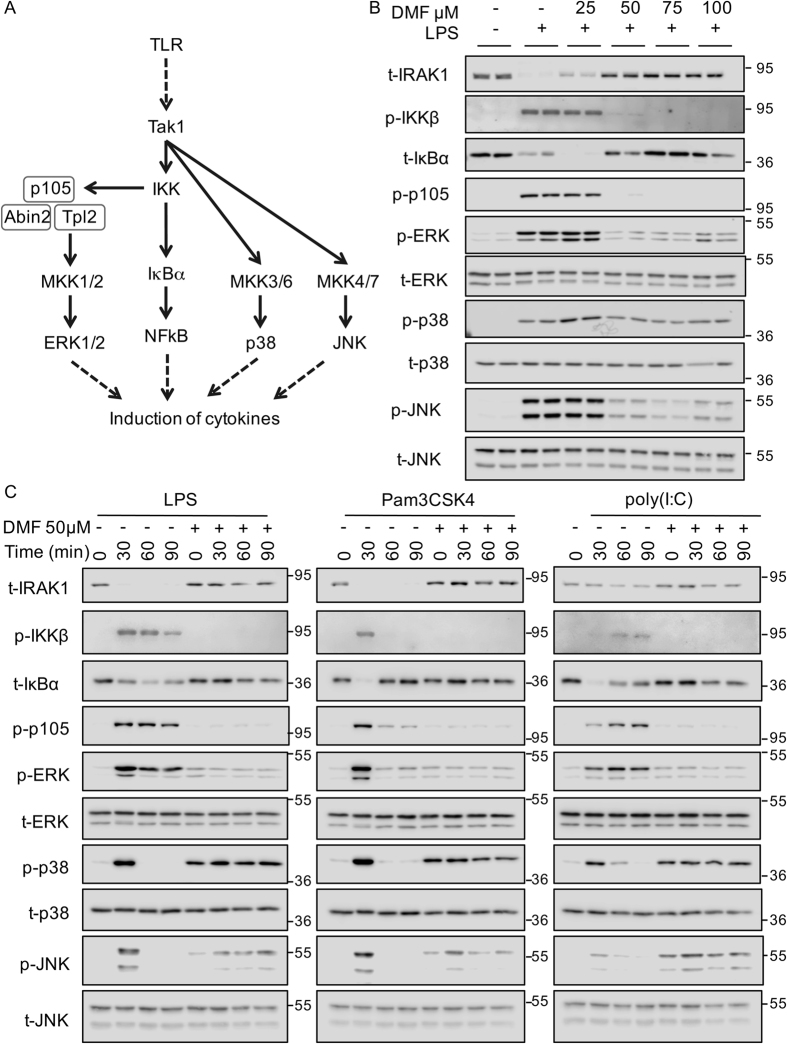
DMF inhibits multiple signals downstream of TLR4. (**A**) TLR4 activates both NFκB and MAPK pathways via Tak1 and IKK in order to drive cytokine production. (**B**) BMDMs were treated with the indicated concentrations of DMF for 4 h. Cells were then stimulated with 100 ng/ml LPS for 30 min. Following cell lysis the levels of the indicated total and phospho proteins were determined by immunoblotting. (**C**) BMDMs were cultured in the presence or absence of 50 μM DMF for 4 h. Cells were then further stimulated for the indicated times with either 100 ng/ml LPS, 1 μg/ml Pam_3_CSK_4_ or 10 μg/ml poly(I:C). The levels of the indicated total and phospho proteins were determined by immunoblotting.

TLR dependent transcription and secretion of cytokines is regulated by the NFκB as well as the ERK1/2 and p38 MAPK signaling pathways^31^. The ability of DMF to block the induction of multiple LPS induced genes (Figs 1 and 2) suggested that it might have a suppressive effect on both MAPK and NFκB activation in response to the TLR4 agonist LPS. TLR4 signals via both MyD88 and Trif dependent pathways, which are thought to converge on the activation of Tak1^31^,^32^,^33^,^34^. Tak1 in turn activates a complex of IKKβ, IKKα and NEMO. IKKβ then activates the classical NFκB pathway via the phosphorylation of IκBα. In addition IKKβ also phosphorylates p105, and this is required for the activation of Tpl2, which is then able to activate the ERK1/2 pathway. Tak1 also directly activates the MKKs required for the activation of the p38 and JNK pathways (Fig. 5A)^31^,^32^,^33^,^34^.

DMF was able to inhibit the activation of the classical NFκB pathway, as 50 μM of DMF was sufficient to block the degradation of IκBα in response to 30 min stimulation with LPS (Fig. 5B). In addition, 50 μM DMF blocked the phosphorylation of IKKβ on Ser177 and 181 (Fig. 5B), sites that correlate with its activation^35^. In agreement with the loss of IKKβ activity, DMF also blocked the phosphorylation of the IKKβ substrate p105 and inhibited the activation of ERK1/2, as judged by phosphorylation on its TXY activation motif. DMF treatment also resulted in a partial inhibition of JNK activation, that was maximal at 75 μM DMF. In contrast, DMF did not inhibit the activation of p38 at any of the concentrations tested. In the MyD88 pathway, IRAKs are involved in propagating the signal from MyD88 to Traf6 and then Tak1. During this process IRAK1 becomes modified with K63 polyubiquitin chains, resulting in the disappearance of the IRAK1 band at its predicted molecular weight on immunoblots that can be reversed by treatment of the lysates with deubiquitinating enzymes^36^,^37^. Interestingly, the loss of the IRAK1 band was partially reversed by 25 μM DMF and completely reversed by 50 μM DMF suggesting that DMF may interfere with IRAK1 ubiquitination (Fig. 5B).

To examine this process in more detail, cells were incubated in 50 μM DMF and then a time course of LPS stimulation carried out (Fig. 5C). As in the previous experiment, DMF inhibited the loss of IRAK1 and IκBα, blocked the phosphorylation of IKKβ and p105 and greatly reduced the phosphorylation of ERK1/2. The effect on JNK phosphorylation was more complex. DMF reduced the phosphorylation of JNK at 30 min. In the absence of DMF, JNK phosphorylation was transient and not observed at 60 or 90 min. In contrast, JNK phosphorylation was still observed at 60 and 90 min of LPS stimulation in the presence of DMF.

LPS has the ability to signal via both the MyD88 and Trif adaptors. To determine if the effects of DMF on signaling were common to pathways downstream of both adaptors, cells were stimulated with either Pam_3_CSK_4_ or poly(I:C). Pam_3_CSK_4_ stimulates TLR1/2 and acts via MyD88 and not Trif. The effects of DMF on Pam_3_CSK_4_ induced signaling mirrored the results seen with LPS (Fig. 5C). Poly(I:C) activates TLR3 and this utilizes Trif and not MyD88 for downstream signaling. Consistent with this poly(I:C) did not strongly promote the ubiquitination of IRAK1. Poly(I:C) was able to induce both IKKβ and p105 phosphorylation and this was blocked by DMF. In addition DMF blocked poly(I:C) induced ERK1/2 activation, however, as for LPS and Pam_3_CSK_4_ there was little effect on p38 activation (Fig. 5C). Poly(I:C) was only a weak activator of JNK, and this activation was slightly increased in the presence of DMF.

**Figure 6:**
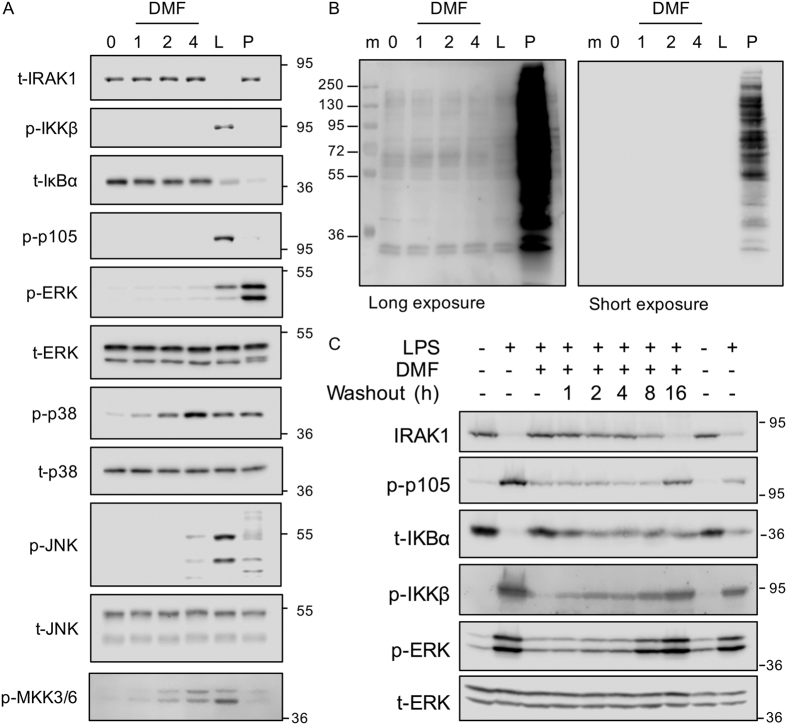
DMF can activate p38 MAPK and can covalently modify its targets in the cell. BMDMs were incubated in 50 μM DMF for the indicated times. Alternatively BMDMs were stimulated with 100 ng/ml LPS for 30 min (L) or 60 μM sodium pervanadate for 10 min (P). (**A**) The levels of the indicated total and phospho proteins were determined by immunoblotting. (**B**) Lysates from A were immunoblotted for p-Tyr. Molecular weights (kDa) are indicated on the left and two exposures are shown. (**C**) Where indicated BMDMs were treated with 50 μM DMF for 4 h. Cells were then washed 3 times to remove the DMF and incubated for the indicated washout times. Finally cells were stimulated with 100 ng/ml LPS for 30 min. The levels of the indicated total and phospho proteins were determined by immunoblotting.

Unexpectedly in these experiments, incubation in DMF alone induced the phosphorylation of p38 and, to a lesser extent, JNK in the absence of any other stimuli (Fig. 5C, lane 5). To examine this further a time course of DMF treatment alone was carried out. DMF treatment in the absence of any additional TLR stimulation did not affect IRAK1 ubiquitination or p105 phosphorylation (Fig. 6A). DMF treatment did however induce phosphorylation of p38, and to a lesser extent JNK, over time. Tyrosine phosphatase inhibitors have previously been found to activate MAPK signaling in cells^38^. Tyrosine phosphatases possess an active site cysteine and may therefore be a target for electrophilic drugs such as DMF^39^. Treatment with the tyrosine phosphatase inhibitor pervanadate induced the phosphorylation of p38 and JNK although, in contrast to DMF, pervanadate also strongly induced ERK1/2 phosphorylation (Fig. 6A). As would be expected pervanadate strongly induced global tyrosine phosphorylation levels. In contrast, DMF had little effect on global tyrosine phosphorylation levels indicating that it does not act as a general tyrosine phosphatase inhibitor in cells (Fig. 6B). MAPKs can be dephosphorylated by DUSPs (Dual Specificity Phosphatases) and inhibition of these enzymes by DMF may account for the p38 and JNK activation. Inhibition of the DUSP would be expected to be specific for the MAPK and not affect the upstream MKK. DMF was found to increase the phosphorylation of MKK3 and 6 suggesting that its effects on p38 and JNK phosphorylation were independent of DUSP inhibition (Fig. 6A).

DMF has the ability to covalently modify cysteine residues in its targets, as is proposed for Keap1. We assessed whether DMF inhibition remained following washout of DMF from the cells. BMDMs were treated for 4 h with 50 μM DMF and then either directly stimulated with LPS or washed extensively and incubated in media without DMF for various times. This showed that signaling was still inhibited by DMF 4 h after DMF being washed off the cells, but that inhibition was lost by 16 h (Fig. 6C). This slow loss of inhibition would be consistent with DMF covalently modifying its target in cells.

*In vivo* DMF can be converted to MMF. We therefore checked if MMF was able to mimic the effects of DMF on cell signaling. Unlike DMF, MMF did not inhibit LPS induced ERK1/2 or p105 phosphorylation, or the ubiquitination of IRAK1 (Supplementary Figure 1).

### DMF inhibits E2 conjugating enzymes *in vitro*

**Figure 7:**
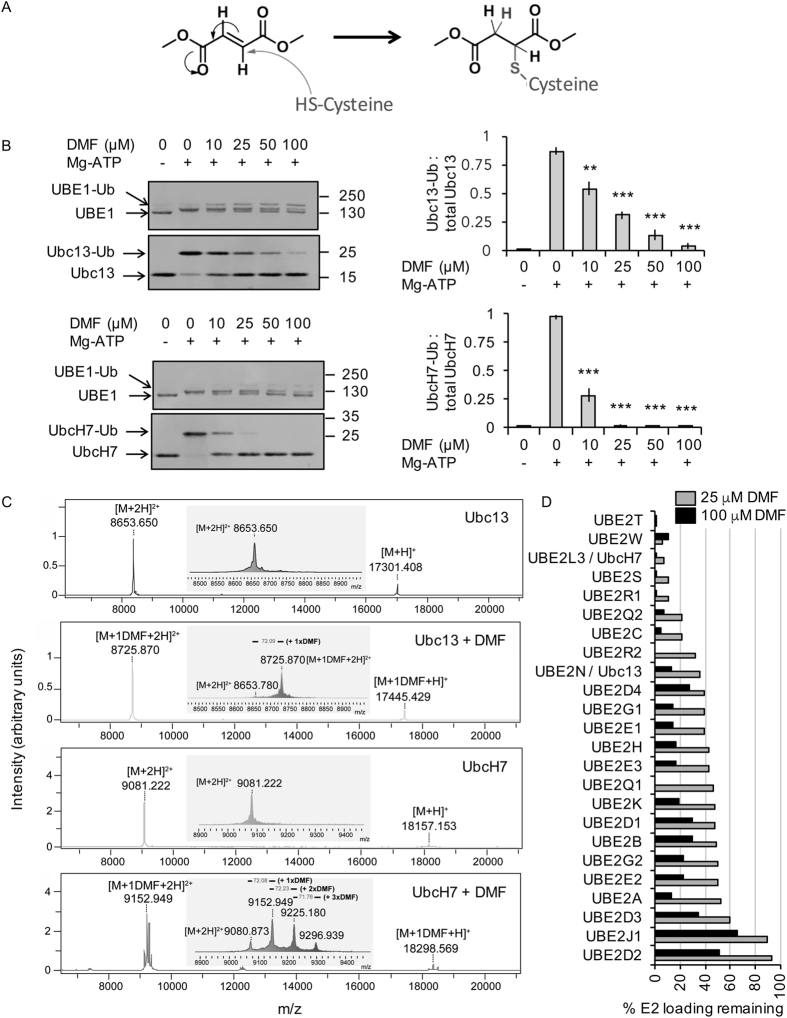
DMF can inhibit E2 conjugating enzymes *in vitro*. (**A**) Reaction mechanism of DMF and cysteine. (**B**) Ubc13 or UbcH7 was incubated in the presence or absence of DMF for 30 minutes. E2 loading reactions for Ubc13 and UbcH7 in the presence of ubiquitin, Ube1 and Mg-ATP were carried out in the presence of increasing concentrations of DMF as described in the methods. Ubiquitin loading was resolved on 4-12% polyacrylamide gels. Example gels are shown on the left and the quantification of 3 separate reactions shown on the right. Error bars represent standard deviation. For DMF treated conditions, a p value of less than 0.001 (Students t-test) is indicated by ***. (**C**) Ubc13 or UbcH7 was incubated in the presence or absence of 50 μM DMF for 4 h. The molecular mass was then determined by MALDI-TOF mass spectrometry. Spectra show intensity vs. m/z and inserts show an expansion of the 2+ peak. (**D**) The indicated E2 conjugating enzymes were incubated with either 25 or 100 μM DMF for 30 minutes and E2 loading assays carried out as described in the methods. The % E2 loading relative to the 0 μM condition for each E2 tested is shown.

Together these results suggest that DMF may act at an upstream point in the TLR signaling cascade. The activation of Tak1 and the recruitment of the IKK complex involves the formation of K63/M1 hybrid polyubiquitin chains which can be added to several proteins in the MyD88 signaling cascade including MyD88 and IRAKs^34^,^40^. The formation of polyubiquitin chains requires a cascade of three enzymes; an E1 activating enzyme, an E2 conjugating enzyme and an E3 ligase to mediate the transfer of the ubiquitin to the substrate. E2 enzymes use a cysteine in their active site to covalently couple to ubiquitin. DMF is an electrophilic compound and therefore has the potential to react with the SH group in cysteines (Fig. 7A). We therefore investigated if DMF could inhibit Ubc13 or UbcH7, the major E2 enzymes proposed to be involved in the formation of K63 and M1 chains^41^,^42^,^43^,^44^,^45^. *In vitro*, addition of the E1 UBE1, ubiquitin and Mg-ATP to an E2 is able to promote the loading of ubiquitin onto the E2, which can be resolved as a mobility shift on SDS polyacrylamide gels^45^. Addition of DMF to this reaction inhibited the loading of ubiquitin onto Ubc13 with an IC50 of between 10 and 20 μM (Fig. 7B). In parallel assays, UbcH7 was more strongly inhibited by DMF with an IC50 of less than 10 μM and complete inhibition by 50 μM (Fig. 7B). If DMF were to inhibit Ubc13 or UbcH7 via covalent modification of a cysteine, the molecular mass of the E2 should be increased following DMF treatment. MALDI-TOF mass spectrometry was therefore used to determine the molecular mass of recombinant Ubc13 and UbcH7 before and after treatment with DMF. The recombinant Ubc13 has a theoretical mono-isotopic mass of 17295 Da, which corresponded well with the observed mass of 17301 Da. For Ubc13, an increase in the molecular mass of 144 Da was seen following incubation in DMF, which would be consistent with the labeling of a cysteine residue in the protein with DMF via a Michael addition reaction (Fig. 7A). For unmodified UbcH7 a mass of 18157 Da was observed relative to a theoretical mono-isotopic mass of 18149 Da. Following DMF treatment of UbcH7 3 peaks were obtained. The major peaks indicated an addition of 144 and 289 Da, which would correspond to addition of 1 or 2 DMF molecules respectively. A minor peak was also obtained with a mass increase of 432 Da, which would suggest addition of 3 molecules of DMF (Fig. 7C). Of note, Ubc13 only contains the active site cysteine whereas UbcH7 also contains 2 further cysteines in addition to its reactive site residue. To further test the ability of 50 or 100 μM DMF to block E2 activity, it was tested against a panel of 24 different E2 enzymes (Fig. 7D). This showed that while DMF could inhibit all the E2’s tested to some extent, it did exhibit some selectivity with UbcH7, Ube2W, Ube2T, Ube2S and Ube2R1 being most strongly inhibited.

### DMF blocks the formation of M1 and M1/K63 polyubuititin chains downstream of TLR signaling

**Figure 8:**
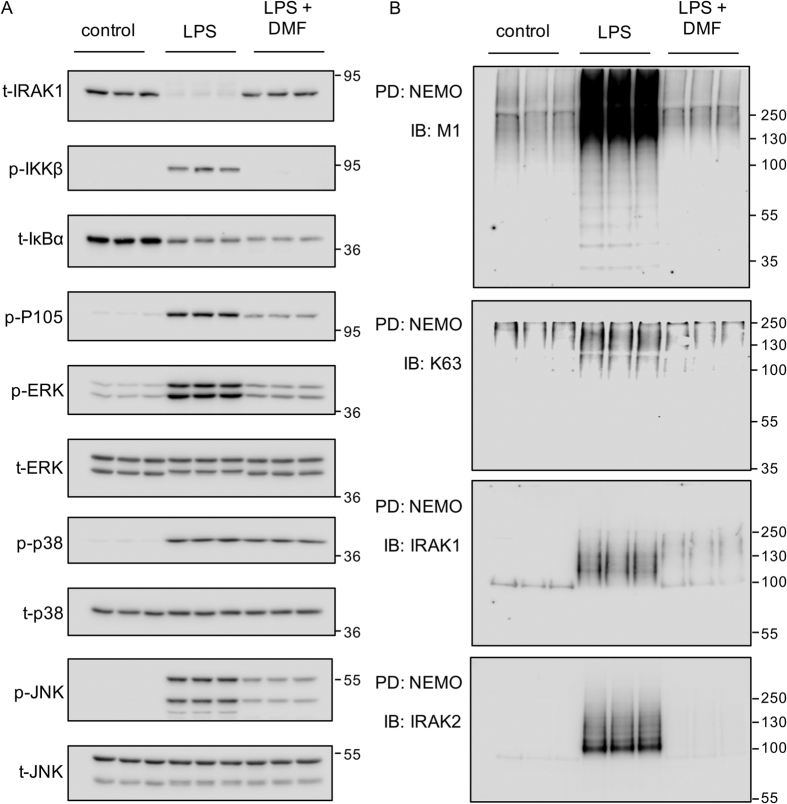
Effect of DMF on LPS stimulated polyubiquitin chain formation in RAW264.7 macrophages. RAW264.7 cells were incubated in the presence or absence of 50 μM DMF for 4 h. Cells were then either left unstimulated or treated with 100 ng/ml LPS for a further 30 min. Cells were lysed and lysates were blotted for the phospho and total proteins indicated (**A**). Alternatively, Halo-NEMO beads were used to pulldown M1 polyubiquitin chains. Pull downs were then immunoblotted with antibodies specific for M1 chains, K63 chains, IRAK1 or IRAK2. Total ubiquitin was pulled down using Halo-TUBE beads and blotted for the presence of K48 chains (**B**).

**Figure 9:**
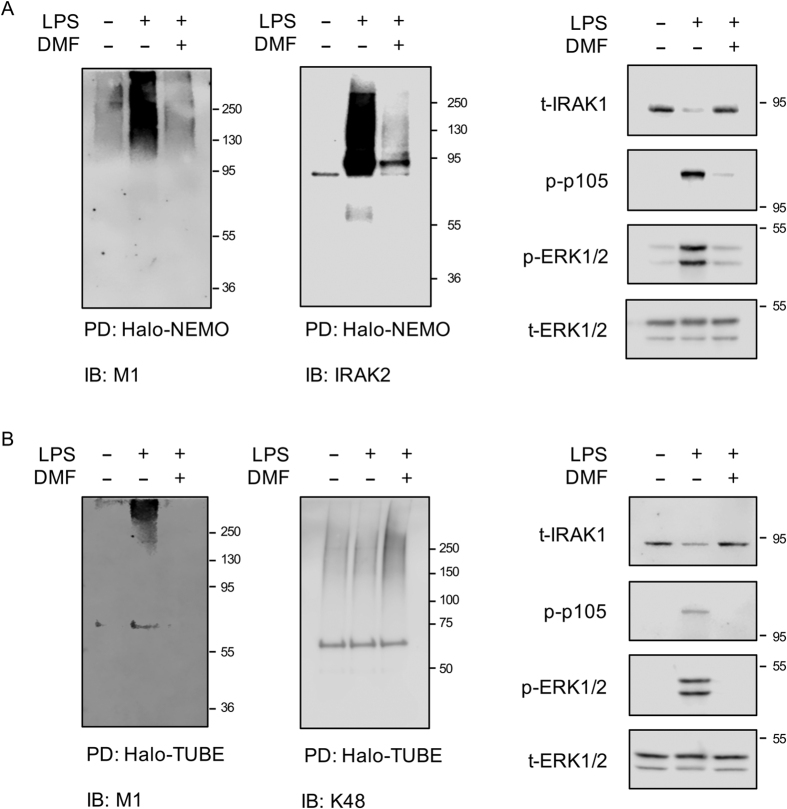
Effect of DMF on LPS stimulated polyubiquitin chain formation in BMDMs. (**A**) BMDMs were incubated in the presence or absence of 50 μM DMF for 4 h. Cells were then either left unstimulated or treated with 100 ng/ml LPS for a further 30 min. Halo-NEMO beads were used to pulldown M1 polyubiquitin chains. Pull downs were then immunoblotted with antibodies specific for M1 chains or IRAK2 (left hand panels). Alternatively the levels of IRAK1, phospho p105, phospho ERK1/2 and total ERK1/2 in the cell extracts were determined by immunoblotting (right hand panels). (**B**) As (**A**) but Halo-TUBE pull downs were used to isolate total ubiquitin chains and the pull downs blotted with antibodies against M1 and K48 chains.

Previous studies have indicated that TLR agonists can stimulate the formation of K63/M1 hybrid polyubiquitin chains^40^,^46^. As, *in vitro*, DMF inhibited Ubc13 and UbcH7, the E2 conjugating enzymes proposed to be involved in the formation K63 and M1 chains respectively, its ability to block formation of M1 and K63 chains in cells was determined. M1-Ub chains were pulled down from RAW264.7 macrophage cell lysates using Halo-tagged NEMO beads, which shows >50 fold selectivity for M1 relative to K63 chains^40^. This approach would be expected to pull down M1-Ub chains, complexes containing both M1 and K63 chains as well as M1/K63 hybrid chains that are proposed to be formed downstream of TLR signaling^40^,^46^. As seen in primary macrophages following TLR stimulation, DMF inhibited the ubiquitination of IRAK1 and the phosphorylation of ERK1/2, p105 and IKK in RAW264.7 cells (Fig. 8A). Halo-NEMO pulldowns were blotted for M1 or K63 polyubiquitin chains on IRAK1 and IRAK2. LPS increased the amount of M1 and M1/K63 hybrid chains in the pulldowns, and this increase was inhibited by pretreatment of the cells with DMF (Fig. 8B). Polyubiquitinated IRAK1 and IRAK2 could also be seen in the pulldowns from LPS stimulated but not untreated cells, and this was blocked by pretreatment with DMF (Fig. 8B). DMF did not however block all ubiquitination within the cell. Halo-TUBE (Tandem Ubiquitin Binding Entities) beads were used to pull down all ubiquitin chains within the cells. Blotting of these pulldowns showed that LPS did not increase the total amount of K11, K48 or K63 linked polyubiquitin chains in these pull downs and that these levels were also not reduced by DMF (Supplementary Figure 2). Similarly using Halo-TAB2-NFZ beads to pull down K63 chains showed that neither LPS or DMF affected the amount of K63 chains in these pull downs (Supplementary Figure 2). This probably reflects a high background level of K63 and K48 chains within the cell so that the changes induced by TLR signaling are too small to be seen. In line with this, blotting the Halo-TUBE or Halo-TAB2-NFZ pull downs for IRAK2 showed that LPS did induce the ubiquitination of this protein and that this was blocked by DMF (supplementary Figure 2). In line with the results in RAW264.7 cells, DMF also greatly reduced the amount of M1 chains that could be pulled down from BMDM lysates using either Halo-NEMO or Halo-TUBE beads (Fig. 9). Similarly the amount of ubiquitinated IRAK2 that came down in the Halo-NEMO pulldowns was also reduced by DMF. In contrast, DMF did not reduce the amount of K48 linked chains in Halo-TUBE pulldowns from BMDMs (Fig. 9).

M1 chains are formed by the LUBAC complex which contains HOIP, HOIL and Sharpin. In this complex HOIP acts as the E3 and directly conjugates to ubiquitin via a cysteine during ubiquitin transfer^47^. As it is possible that DMF might inhibit HOIP via reacting with this cysteine in cells, HOIP was immunoprecipitated from cells and its activity examined in an *in vitro* ubiquitination reaction. Due to the covalent modification of the cysteine by DMF, inhibition in the cell should be retained during the immunoprecipitation and *in vitro* assay. Consistent with previous reports^40^, HOIP was constitutively active in this assay, and this activity was not affected by pretreatment of the cells with DMF (Supplementary Figure 3).

### DMF inhibits K63/M1 chain formation in response to IL-1 and TNF

**Figure 10:**
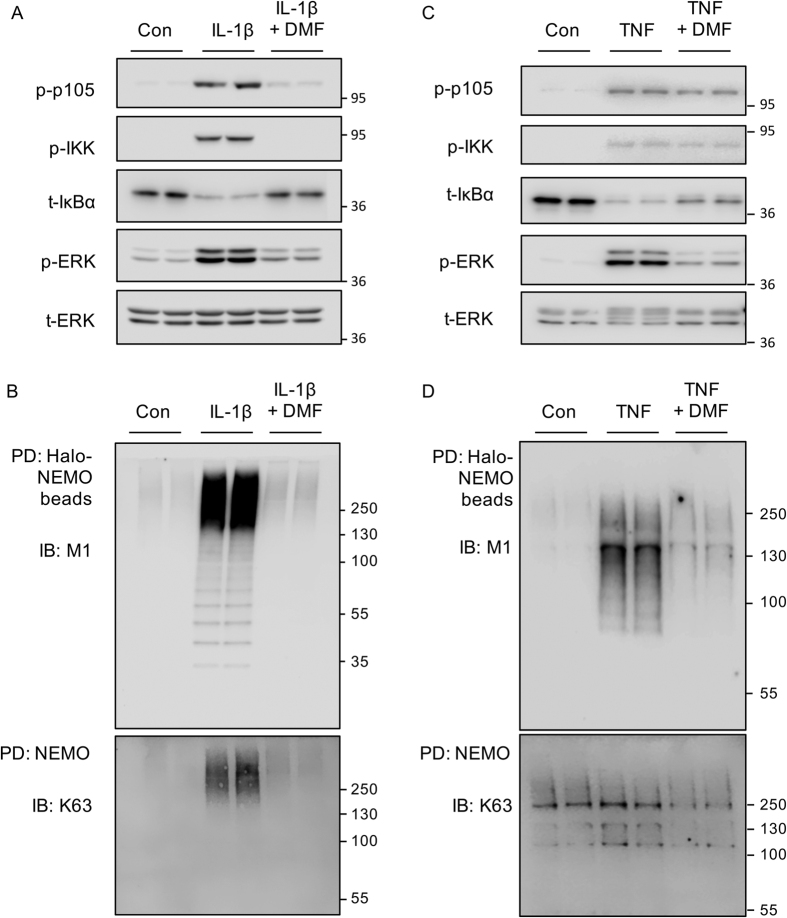
Effect of DMF on IL-1 and TNF stimulated M1 chain formation. (**A**) IL-1R HEK293 cells were incubated with 50 μM DMF for 4 h where indicated. Cells were stimulated with 5 ng/ml IL-1β for 30 min and the levels of phospho p105, phospho IKK, total IκBα, phospho ERK1/2 and total ERK1/2 determined by immunoblotting. (**B**) As (**A**) but Halo-NEMO beads were used to pull down M1 polyubiquitin chains. Pulldowns were blotted for M1 and K63 linked polyubiquitin chains. (**C**) HeLa cells were incubated with 50 μM DMF for 4 h where indicated. Cells were stimulated with 10 ng/ml TNF for 30 min and the levels of phospho p105, phospho IKK, total IκBα, phospho ERK1/2 and total ERK1/2 determined by immunoblotting. (**D**) As (**C**) but Halo-NEMO beads were used to pull down M1 polyubiquitin chains. Pull downs were blotted for M1 and K63 linked polyubiquitin chains.

The formation of K63 and M1 chains is not restricted to TLR signaling and also occurs downstream of other stimuli including IL-1 and TNF^34^,^40^. To examine chain formation downstream of IL-1, HEK293 cells stably expressing the IL-1 receptor were stimulated with IL-1 in the presence or absence of DMF. In these cells IL-1 stimulated the phosphorylation of p105 and IKK as well as the degradation of IκBα which was blocked by DMF, as was the activation of ERK1/2 (Fig. 10A). In Halo-NEMO pulldowns, IL-1 stimulation increased the amount of both M1 and K63 chains observed, and this was blocked by pretreatment of the cells with DMF (Fig. 10B). Treatment of HeLa cells with TNF induced ERK1/2, IKK and p105 phosphorylation as well as the degradation of IκBα. ERK1/2 phosphorylation and IκBα degradation were inhibited by DMF, however in these cells DMF did not block p105 or IKK phosphorylation in response to TNF (Fig. 10C). NEMO pulldowns showed that TNF stimulated the formation of M1 polyubiquitin chains, and this was reduced by DMF. TNF only resulted in a minor increase in the signal for K63 chains, but again this was reduced by DMF (Fig. 10D).

## Discussion

While the ability of DMF to modulate immune function is well established, its mechanism of action has been more elusive, and several potential *in vivo* targets have been suggested. While DMF can activate the Nrf2 anti-oxidant pathway, our results, along with those recently published by Gillard *et al.*^48^, indicate that this is not required for the ability of DMF to block cytokine induction. In our hands DMF has a suppressive effect on several pathways downstream of TLR signaling, including NFκB and ERK1/2 signaling. We demostrate here that DMF can inhibit cytokine production in macrophages and that this correlates to the inhibition of NFκB and MAPK signaling pathways. This would suggest that DMF acts at an upstream point in TLR signaling, and in line with this we show that DMF can block the formation of signaling complexes containing M1 and K63 polyubiquitin chains downstream of TLR and IL-1 signaling. Furthermore, *in vitro* DMF can inhibit UbcH7 and Ubc13, the major E2 enzymes thought to be required for K63 and M1 chain formation in response to IL-1 or TLR agonists. DMF did not result in a global loss of polyubiquitin chains. Notably the levels of K63 and K48 chains in pull downs with Halo-TUBE beads was unaffected by DMF. One explanation is that the majority of these chains were preformed in the cell prior to the addition of DMF. If the turnover of the chains was slow, no effect of DMF would be expected on the levels of these chains. In line with this, LPS did not increase the levels of either K48 or K63 chains in the Halo-TUBE pulldowns indicating the presence of a significant number of K48 and K63 cells in resting cells. These observations does not mean TLR signaling cannot promote the K48 or K63 linked ubiquitination of specific proteins only that any such modifications do not measurably alter the total amount of K48 and K63 chains in the cell. TLR signaling has been shown to promote K63 and M1 polyubiquitin chain formation on a number of signaling proteins including IRAK1. Previous reports have indicated that many of these chains actually from as hybrids of K63 and M1 chains^40^,^46^, and in line with this we see increased amounts of K63 and M1 chains in pull downs with Halo-NEMO beads, which selectively pulls down complexes containing M1 chains. The TLR induced formation of these K63 and M1 chains was blocked by pretreatment with DMF (Figs 8, 9, 10).

DMF is converted to MMF *in vivo*. The effects we observed on signaling were specific for DMF, as we did not observe any effect of MMF on LPS induced signaling. This is in agreement with a previous study showing that DMF, but not MMF, repressed TLR induced cytokine induction in primary human PBMCs^48^. In macrophages we show that DMF blocked the activation of the IKK complex and thus prevented both p105 phosphorylation and IκBα degradation. Previous reports have also indicated that DMF may inhibit NFκB transcriptional activity. For example, DMF reduced NFκB activity in LPS stimulated dendritic cells and TNF stimulated HUVEC or ASMC^13^,^14^,^15^. A common feature of these earlier studies was that DMF inhibited NFκB DNA binding or relocalization to the nucleus, but did not have a major effect on the degradation of IκBα. The reason for the difference between these studies and our findings is not clear. One potential explanation is the different cell types and stimuli used in the various studies may influence the results seen for IκBα degradation. In line with this, the ability of DMF to inhibit IκBα degradation in TNF stimulated HeLa cells was much less than in TLR stimulated macrophages. Another issue could relate to how DMF was used. The concentrations of DMF were similar in the different studies, however the pre-incubation times in these previous papers were not clear and were likely different to the 4 h preincubation used in our studies. As DMF can act as a covalent inhibitor it is able to display time dependent kinetics of inhibition (data not shown) and thus longer pre-incubation times might give a more pronounced inhibition of IκBα degradation.

The effects we observed on NFκB and MAPK activation could be explained by inhibition of TLR signaling at the level of K63/M1 polyubiquitin chain formation and thus blocking the activation of Tak1. If DMF did act at the level of Tak1 or ubiquitination, it would also be expected to inhibit p38 activation. This is complicated by the observation that p38 was activated by DMF alone (Fig. 6), which would obscure any effect of DMF on TLR induced p38 activation pathways. One potential mechanism would be the inhibition of tyrosine phosphatases, as this is known to activate MAPK signaling. This is however unlikely as DMF did not result in increased global levels of phosphotyrosine. Another explanation could be activation of a MAP3K for p38 and JNK. One possible candidate might be Ask1, which is also known to be expressed in macrophages and can contribute to p38 activation by LPS^49^. Ask1 is activated by oxidative stress via a complex mechanism involving Trx1^50^. It is possible that DMF may activate Ask1 via modification of cysteine residues on Trx1 or via targeting proteins involved in the response to oxidative stress. Further work would be required to resolve this point.

The effects of DMF on signaling have recently been suggested to have similarities to those produced by BAY 11-7082^48^. Interestingly while BAY 11-7082 was originally described as an IKK inhibitor a recent study has shown that BAY 11-7082 can act in cells via inhibiting E2 enzymes, including Ubc13 and UbcH7^45^. As for DMF, the mechanism found for this involved the covalent modification of the active site cysteine in the E2.

Taken together our results, along with those of others, would indicate that DMF has complex effects in cells and is unlikely to act via a single target. Instead it is likely that the overall therapeutic effects of DMF in MS or psoriasis are due to the ability of DMF to directly modify the function of a number of cellular proteins via the modification of reactive cysteines in these targets. The finding that DMF - an approved drug in clinical use - and BAY 11-7082 can both target E2s involved in K63 and M1 polyubiquitin chain formation and have similar effects on cytokine production suggests that E2s may be useful drug targets for novel anti-inflammatory therapies.

## Methods

### Chemicals

All chemicals, including DMF and MMF, were from Sigma unless otherwise stated.

### Animals

C57Bl/6 mice were obtained from Charles River Laboratories. Nrf2 knockout mice have been described previously^51^ and backcrossed onto C57Bl/6 for 7 generations^52^. Knockouts of MSK1 and MSK2 have been described previously and mice used in these studies had been backcrossed onto C57Bl/6 for at least 20 generations^53^. Animals were maintained in individually ventilated cages under specific pathogen free conditions. Mice were maintained in accordance with UK and EU regulations, and all protocols were covered by an appropriate Home Office license that was subject to approval by the University of Dundee Ethical Review Committee.

### Cell culture

All cell culture reagents were from Gibco unless otherwise stated. RAW264.7 cells were maintained in DMEM supplemented with 10% heat-inactivated fetal bovine serum (FBS), 2 mM L-glutamine, 100 units/ml penicillin G, 100 μg/ml streptomycin. HEK293 cells stably overexpressing the IL-1 receptor^54^ and HeLa cells were maintained in DMEM supplemented with 10% FBS, 2 mM L-glutamine, 100 units/ml penicillin G, 100 μg/ml streptomycin. Bone marrow derived macrophages (BMDMs) were isolated as described^55^. Briefly, freshly isolated bone marrow cells were differentiated in bacterial-grade plastic dishes in DMEM supplemented with 10% heat inactivated fetal bovine serum (Labtech), 4 mM L-glutamine, 100 units/ml penicillin G, 100 μg/ml streptomycin, 0.25 μg/ml amphotericin and 5 ng/ml M-CSF (PeproTech). After 7 days macrophages were passaged onto tissue culture plastic and used the following day. Bone marrow derived dendritic cells (BMDCs) were prepared in a similar manner to BMDMs but 5 ng/ml GM-CSF (Peprotech) was used in place of M-CSF.

### Analysis of cytokine induction

Following stimulation, the levels of TNF, IL-6, IL-10, IL-13 and GM-CSF present in the media was determined via a multiplex Luminex based method (Bioplex, BioRad) according to the manufacturer’s instructions. For measurement of mRNA induction, total RNA was isolated using the Omega MicroElute total RNA extraction kit (VWR). 0.5 to 1 μg of total RNA was reversed transcribed (iScript, BioRad) and qPCR carried out using Sybrgreen based detection reagents (SYBR Premix ExTaqII #RR820L, Takara Biosciences). Fold induction was calculated from the unstimulated controls cells and GAPDH was used to correct for expression^56^. Primer sequences are shown in Table 1.

### Flow cytometry

BMDMs were treated with 50 μM DMF. To assess TNFα induction, cells were subsequently stimulated with 100 ng/ml LPS to induce cytokine, and cytokine secretion was blocked by treating cells with 3 μg/ml Brefeldin-A and 2 μM Monensin. Cells were detached by treatment with 1x trypsin for 5 min and gentle scraping. Cells were washed with PBS and the percentage of live cells was determined by staining with Zombie-Aqua (BioLegend) according to the manufacturer’s protocol. Briefly, cells were resuspended in 1:200 Zombie-Aqua in PBS and were incubated at room temperature in the dark for 10 minutes. Then, cells were washed with FACS buffer (1% BSA in PBS), fixed with Fixation buffer (eBiosciences) for 20 min at +4 °C, washed with FACS buffer, permeabilised with 1x Permeabilisation buffer (eBiosciences) for 15 min at +4 °C and washed with FACS buffer. Cells were incubated with 1:100 Fc block (BD Biosciences) in 1x permeabilisation buffer for 10 min at +4 °C, and then stained for intracellular TNFα with TNFα-PE (eBiosciences) in 1x permeabilisation buffer for 20 min at +4 °C. Stained cells were washed with FACS buffer, resuspended in FACS buffer and acquired in a BD FACSCanto™ II.

### Immunoblotting

Cells were lysed in 50 mM Tris-HCl (pH 7.5), 1 mM EGTA, 1 mM EDTA, 1 mM sodium orthovanadate, 50 mM sodium fluoride, 1 mM sodium pyrophosphate, 0.27 M sucrose, 1% (vol/vol) Triton X-100, 0.1% (vol/vol) 2-mercaptoethanol, 1 μg/ml aprotinin, 1 μg/ml leupeptin, 1 mM PMSF. Lysates were clarified by centrifugation (13,000 rpm for 10 min at 4 °C) and supernatants snap-frozen and stored at −80 °C. Protein concentration was determined with Coomassie Protein Assay Reagent (Thermo Scientific). Proteins were separated on 10% polyacrylamide gels and immunoblotting carried out using standard techniques. Antibodies recognizing phospho-Thr202/Tyr204 ERK (#9101), total ERK (#9102), total JNK (#9258), phospho-Thr180/Tyr182 p38 (#4511), total p38 (#9212), total IRAK1 (#4504), phospho-S133 P105 (#4806), total IκBα (#4814), phospho-MKK3/MKK6 (#9321) and phospho-Ser177/Ser181 IKKβ (#2697) were from Cell Signaling Technology. A phospho-specific antibody that recognizes JNK phosphorylated at Thr183/Thr185 (#44682) was from Invitrogen. An antibody recognizing IRAK2 (#62419) was from Abcam. An antibody recognizing ubiquitin was from Dako (#Z0458), and antibodies recognizing Lys63-linked poly-ubiquitin (#HWA4C4), Lys-48-linked poly-ubiquitin (#05-1307/Apu2) and Lys-11-linked poly-ubiquitin (2A3/2E6) were from Merck-Millipore. The antibody recognizing linear M1-linked poly-ubiquitin has been described^57^. The antibody recognizing HOIP (S174D 4^th^ bleed) was raised in sheep and purified by the Antibody Production Team of the DSTT, Medical Research Council Protein Phosphorylation and Ubiquitylation Unit, University of Dundee.

### E1 and E2 ubiquitin loading assays

UbeE1 and E2s were expressed and purified as reported previously^45^. E1 and E2 ubiquitin loading assays were performed as described^45^. Briefly, UBE1 (0.17 μM) was incubated with Ubc13 (2.4 μM) or UbcH7 (2.9 μM) and 10 μM ubiquitin in 20 mM HEPES pH 7.5 with or without 1 μl DMF for 30 minutes at room temperature. A solution of 10 mM magnesium acetate and 0.2 mM ATP was added, reactions incubated at 30 °C for 10 minutes, and terminated by the addition of 5 μl 4x NuPAGE LDS sample buffer. Samples were subjected to SDS-PAGE in the absence of thiol. Gels were stained for 1 h with Coomassie Instant Blue (Expedeon) and destained in water, before imaging and quantification using Licor Odyssey and ImageStudio software.

### LUBAC E3 ligase activity assay

LUBAC E3 ligase activity assays were performed as described^40^. Briefly, 1 μg anti-HOIP was coupled to 10 μl packed Protein-G sepharose beads in 50 mM Tris-HCl pH 7.5, 0.2% Triton X-100 for 2 h at 4 °C. The beads were washed 3x in lysis buffer and incubated with 1 mg cell extract for 16 h at 4 °C. The beads were washed 3x in 50 mM Tris-HCl pH 7.5, 1% Triton X-100, 0.2 M NaCl, 0.05% (v/v) 2-mercaptoethanol and once in 50 mM Tris-HCl pH 7.5, 5 mM MgCl_2_. The E3 ligase reaction was initiated by the addition of 20 μl of 20 mM Tris-HCl pH 7.5, 2 mM DTT, 0.1 μM UBE1, 0.4 μM UbcH7, 10 μM ubiquitin, 5 mM MgCl_2_, 2 mM ATP and incubated for 60 minutes at 30 °C. The reactions were terminated by the addition of 5 μl 4x NuPAGE LDS sample buffer and samples were subjected to SDS-PAGE and immunoblotting with anti-ubiquitin antibody (DAKO #Z0458).

### Generation of Halo-tagged proteins and pull-down assays

NEMO, TAB2-NFZ domain or tandem-repeated ubiquitin binding entities (TUBEs) were expressed as Halo-tagged proteins in *E. coli* and purified as described^40^. To capture M1-pUb and/or K63-pUb chains from cell extracts, 2–3 mg of cell extract was incubated with 20–30 μl (packed beads) Halo-linked proteins for 16 h at 4 °C. The beads were washed 3x with 50 mM Tris-HCl pH 7.5, 500 mM NaCl, 1% Triton X-100, transferred to Spin-X columns in 600 μl Tris-HCl pH 7.5 and centrifuged twice to remove any supernatant. Proteins were denatured in NuPAGE LDS containing reducing agent, centrifuged to separate them from the beads and subjected to SDS-PAGE and immunoblotting.

### Measurement of the molecular mass of Ubc13 and UbcH7 by MALDI-TOF-MS

Ubc13 (2.4 μM) or UbcH7 (2.9 μM) was incubated with 50 μM DMF in 25 mM HEPES pH 7.5 for 4 h at room temperature, before buffer exchange into 5 mM Tris-HCl pH 7.5. An aliquot of the reaction (2 μl, ~6 pmols) was added to 2 μl of the matrix (2,5-dihydroxyacetophenone, 15 mg/ml in 80% EtOH, 20% 12 mg/ml ammonium citrate bibasic) and 2 μl of 2%(v/v) trifluoroacetic acid was added before spotting 0.5 μl of the sample on to an AnchorChip target (Bruker Daltonics). The analysis was performed manually in reflectron positive mode using a MALDI–TOF mass spectrometer (UltrafleXtreme, Bruker Daltonics). For external calibration, six average masses were used: insulin [M+H]^+^avg (m/z 5734.520), cytochrome c [M+2H]^2+^avg (m/z 6181.050), myoglobin [M+2H]^2+^avg (m/z 8476.660), ubiquitin I [M+H]^+^avg (m/z 8565.760) and cytochrome C [M+H]^+^avg (m/z 12360.970).

## Additional Information

**How to cite this article**: McGuire, V. A. *et al.* Dimethyl fumarate blocks pro-inflammatory cytokine production via inhibition of TLR induced M1 and K63 ubiquitin chain formation. *Sci. Rep.* **6**, 31159; doi: 10.1038/srep31159 (2016).

## Supplementary Material

Supplementary Information

## Figures and Tables

**Table 1:** Primer sequences used for PCR.

mRNA	sense	antisense
GAPDH	ACAGTTCTTATGTGGTGACCC	TGCACCACCAACTGCTTAG
TNF	CAGACCCTCACACTCAGATCATC	GGCTACAGGCTTGTCACTCG
IL-6	TTCCATCCAGTTGCCTTCTTG	AGGTCTGTTGGGAGTGGTATC
IL-10	CCCTTTGCTATGGTGTCCTTTC	GATCTCCCTGGTTTCTCTTCCC
GM-CSF	CTCACCCATCACTGTCACCC	TGAAATTGCCCCGTAGACCC
IL-12p40	TCATCAGGGACATCATCAAACC	TGAGGGAGAAGTAGGAATGGG
IL-23p19	ATCCAGTGTGAAGATGGTTGTGAC	TTCTAGTAGGGAGGTGTGAAGTTG
IκBα	ACACGTGTCTGCACCTAG	TCAGACGCTGGCCTCCAAAC
IFNβ	GGAAAAGCAAGAGGAAAGATTGAC	CCACCATCCAGGCGTAGC
HO-1	CTAGCCTGGTGCAAGATA	GAAGCTGAGAGTGAGGAC
