# Supplementary material for: Dimethyl fumarate blocks pro-inflammatory cytokine production via inhibition of TLR induced M1 and K63 ubiquitin chain formation
Source: Sci Rep. 2016 Aug 8;6:31159. doi: 10.1038/srep31159 (PMC4976367; doi:10.1038/srep31159)
Supplement: Supplementary Information [file srep31159-s1.pdf]

Supplementary data

Dimethyl fumarate blocks pro-inflammatory cytokine production via inhibition of TLR induced M1 and K63 ubiquitin chain formation.

Victoria A. McGuire, Tamara Ruiz-Zorrilla Diez, Christoph H. Emmerich, Sam Strickson, Maria Stella Ritorto, Ruhcha V. Sutavani, Anne Weiβ, Kirsty F. Houslay, Axel Knebel, Paul J. Meakin, Iain R. Phair, Michael L. J. Ashford, Matthias Trost and J. Simon C. Arthur

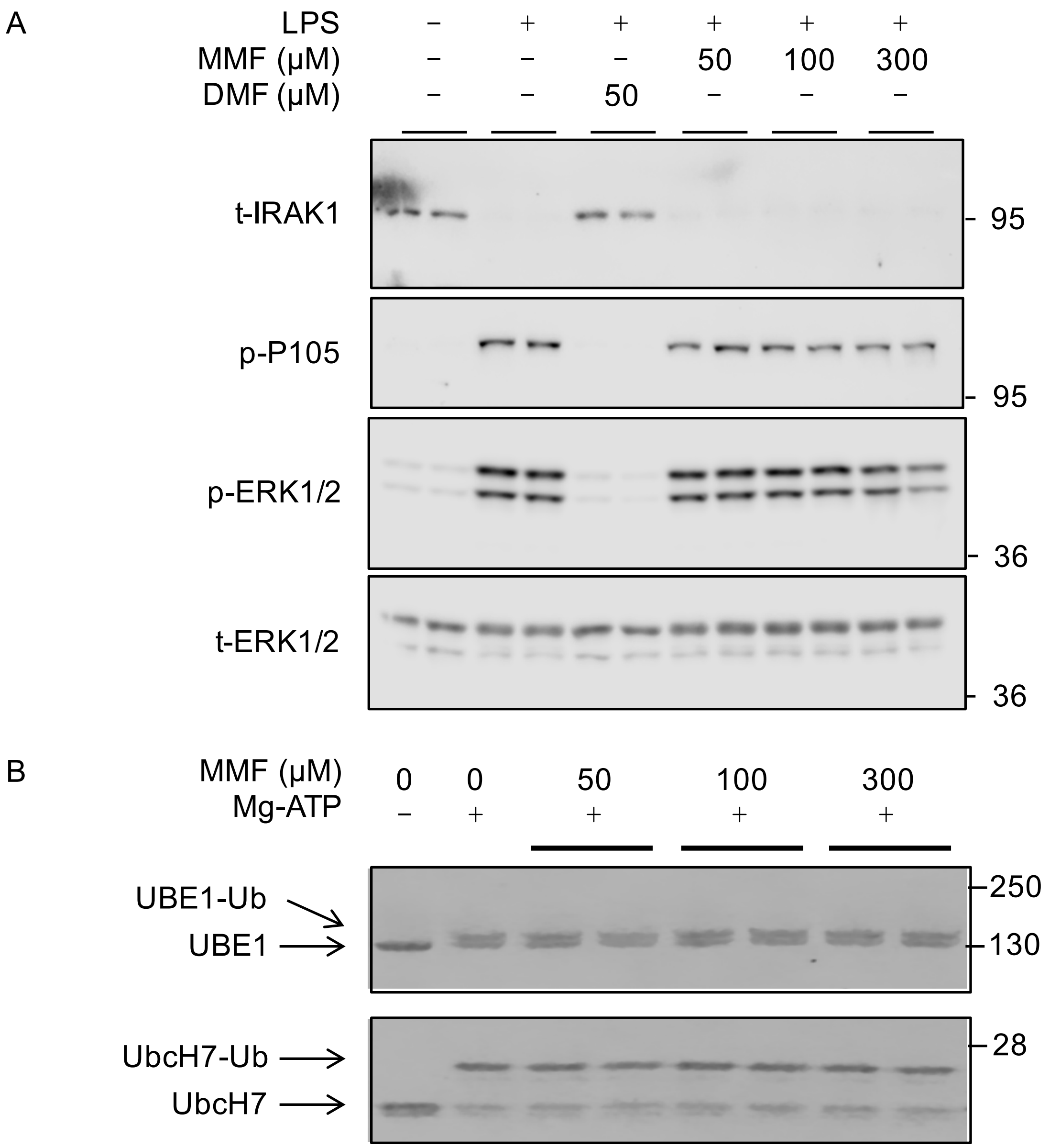

Supplementary Figure 1. MMF doe not mimic the effect of DMF on LPS induced signalling or in vitro loading of UbcH7.

A) BMDMs were incubated in the indicated concentrations of MMF or DMF for 4 h and then cells were stimulated with 100 ng/ml LPS for 30 min. Cells were then lysed and the levels of the indicated proteins determined by immunoblotting.

B) UbcH7 was incubated in the presence or absence of MMF for 30 minutes. E2 loading reactions for Ubc13 and UbcH7 in the presence of ubiquitin, Ube1 and Mg-ATP were carried out in the presence of increasing concentrations of MMF as described in the methods. Ubiquitin loading was resolved on 4-12% polyacrylamide gels.

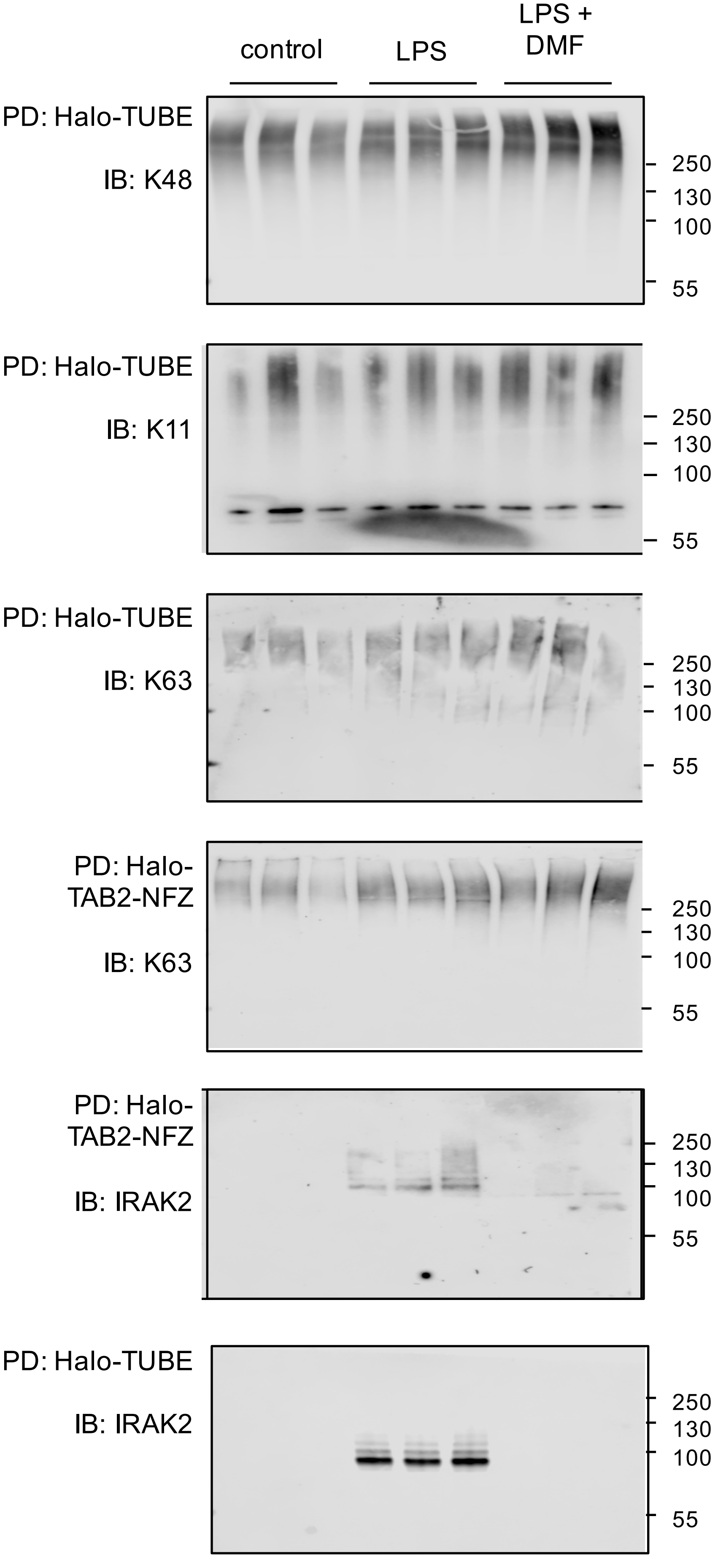

Supplementary Figure 2. DMF does not reduce K48, K63 or K11 chain levels in Halo-NEMO or Halo-TAB2-NFZ pull downs.

RAW264.7 cells were incubated in the presence or absence of 50 µM DMF for 4h. Cells were then left unstimulated or treated with 100ng/ml LPS for 30 minutes. Cells were lysed and pull downs performed with either Halo—NEMO beads, which selectively pull down M1 chains, or Halo-TAB2-NFZ domain beads which pull down K63 linked polyubiquitin chains. Pull downs were then blotted for the indicated polyubiquitin chains or proteins.

-

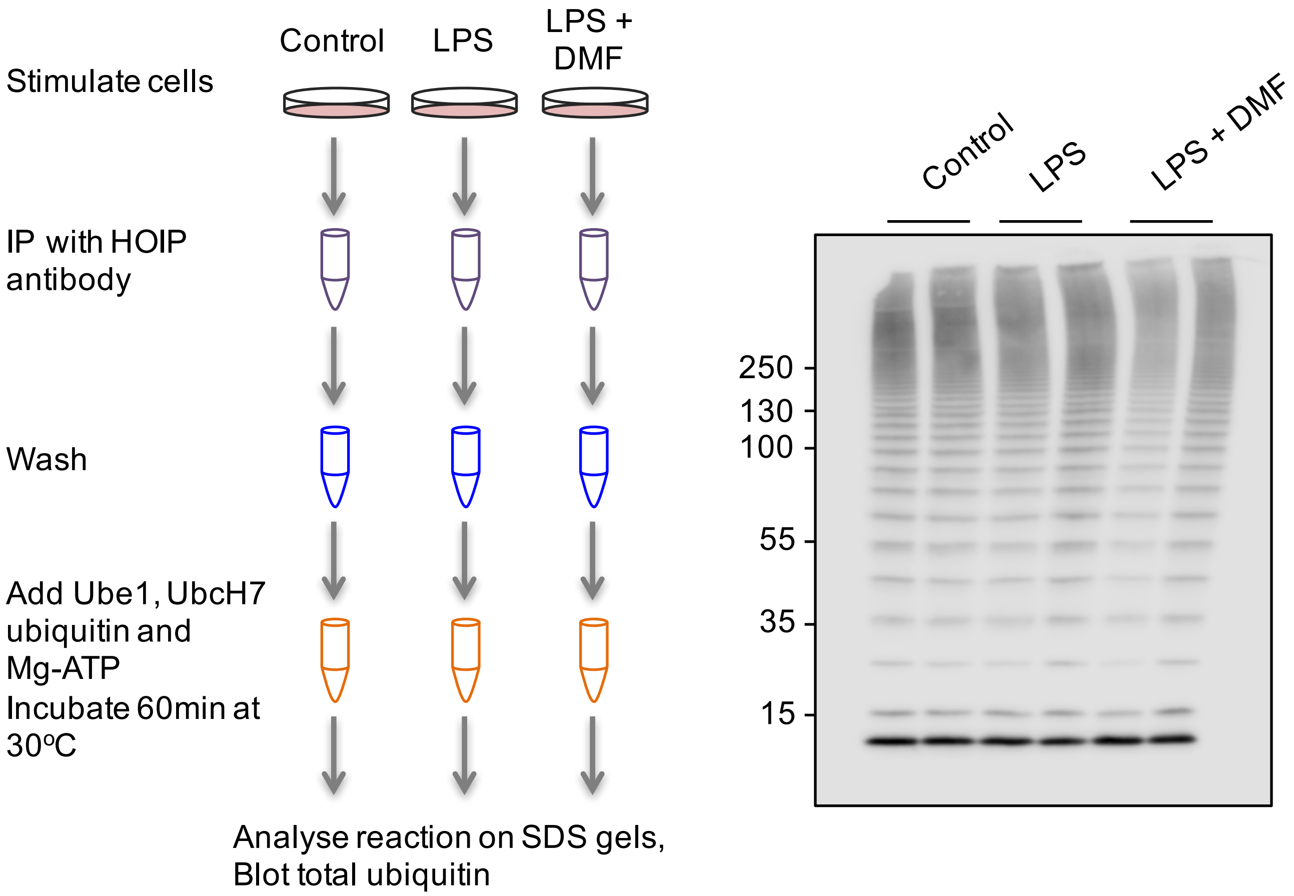

Supplementary Figure 3. DMF does not directly inhibit the LUBAC complex.
RAW264.7 cells were incubated in the presence or absence of 50 μM DMF for 4h. Cells were then left unstimulated or treated with 100ng/ml LPS for 30 minutes. Cells were lysed and LUBAC immunoprecipitated using anti-HOIP antibody as described in the methods. After washing, the E3 ligase assay was initiated by the addition of Ube1, UbcH7, ubiquitin and ATP and analysed by immunoblotting with anti-ubiquitin following SDS-PAGE.
